# Chloroquine and Hydroxychloroquine for the Prevention or Treatment of COVID-19 in Africa: Caution for Inappropriate Off-label Use in Healthcare Settings

**DOI:** 10.4269/ajtmh.20-0290

**Published:** 2020-04-22

**Authors:** Pascale M. Abena, Eric H. Decloedt, Emmanuel Bottieau, Fatima Suleman, Prisca Adejumo, Nadia A. Sam-Agudu, Jean-Jacques Muyembe TamFum, Moussa Seydi, Serge P. Eholie, Edward J. Mills, Oscar Kallay, Alimuddin Zumla, Jean B. Nachega

**Affiliations:** 1 Infectious Diseases Outpatient Clinic, Cameroon and Infectious Diseases Society of Cameroon, Yaoundé, Douala, Cameroon;; 2 Division of Clinical Pharmacology, Department of Medicine, Stellenbosch University, Cape Town, South Africa;; 3 Department of Clinical Sciences, Institute of Tropical Medicine, Antwerp, Belgium;; 4 Discipline of Pharmaceutical Sciences, University of Kwazulu-Natal, Durban, South Africa;; 5 Department of Nursing, University of Ibadan, Ibadan, Nigeria;; 6 Institute of Human Virology and Department of Pediatrics, University of Maryland School of Medicine, Baltimore, Maryland;; 7 International Research Center of Excellence, Institute of Human Virology Nigeria, Abuja, Nigeria;; 8 Department of Paediatrics, University of Cape Coast School of Medical Sciences, Cape Coast, Ghana;; 9 Department of Medical Microbiology and Virology, National Institute of Biomedical Research (INRB), Faculty of Medicine, University of Kinshasa, Kinshasa, Democratic Republic of the Congo;; 10 Service de Maladies Infectieuses et Tropicales, Centre Hospitalo-Universitaire de Fann, Université Cheik Anta-Diop, Dakar, Sénégal;; 11 Unit of Infectious Diseases, Treichville University Teaching Hospital, Abidjan, Côte d’Ivoire;; 12 Unité de Dermatologie et Infectiologie, Unité de Formation et de Recherche, Université Félix Houphouet Boigny, Abidjan, Côte d’Ivoire;; 13 University of Kigali School of Public Health, Kigali, Rwanda;; 14 Erasme Hospital, Free University of Brussels, Brussels, Belgium;; 15 Department of Infection, Division of Infection and Immunity, Centre for Clinical Microbiology, University College London, London, United Kingdom; 16 National Institute for Health Research Biomedical Research Centre, University College London Hospitals, London, United Kingdom; 17 Center for Infectious Diseases, at Stellenbosch University, Cape Town, South Africa;; 18 Department of International Health and Epidemiology, Johns Hopkins Bloomberg School of Public Health, Baltimore, Maryland;; 19 University of Pittsburgh Graduate School of Public Health and Center for Global Health, Pittsburgh, Pennsylvania

## Abstract

The novel severe acute respiratory syndrome-coronavirus-2 pandemic has spread to Africa, where nearly all countries have reported laboratory-confirmed cases of novel coronavirus disease (COVID-19). Although there are ongoing clinical trials of repurposed and investigational antiviral and immune-based therapies, there are as yet no scientifically proven, clinically effective pharmacological treatments for COVID-19. Among the repurposed drugs, the commonly used antimalarials chloroquine (CQ) and hydroxychloroquine (HCQ) have become the focus of global scientific, media, and political attention despite a lack of randomized clinical trials supporting their efficacy. Chloroquine has been used worldwide for about 75 years and is listed by the WHO as an essential medicine to treat malaria. Hydroxychloroquine is mainly used as a therapy for autoimmune diseases. However, the efficacy and safety of CQ/HCQ for the treatment of COVID-19 remains to be defined. Indiscriminate promotion and widespread use of CQ/HCQ have led to extensive shortages, self-treatment, and fatal overdoses. Shortages and increased market prices leave all countries vulnerable to substandard and falsified medical products, and safety issues are especially concerning for Africa because of its healthcare system limitations. Much needed in Africa is a cross-continental collaborative network for coordinated production, distribution, and post-marketing surveillance aligned to low-cost distribution of any approved COVID-19 drug; this would ideally be piggybacked on existing global aid efforts. Meanwhile, African countries should strongly consider implementing prescription monitoring schemes to ensure that any off-label CQ/HCQ use is appropriate and beneficial during this pandemic.

## PERSPECTIVE

Novel coronavirus disease (COVID-19), caused by the novel severe acute respiratory syndrome-coronavirus-2 (SARS-CoV-2), has rapidly spread into a global pandemic. Africa initially appeared spared, but as of this writing, all countries except Lesotho have confirmed cases. As of April 15, 2020, there were 11,367 confirmed COVID-19 cases, with 523 deaths (4.6% case fatality) reported across the WHO African region.^1^ In other settings such as the United States, Europe, and China, morbidity and mortality have been highest in those older than 60 years and with underlying comorbidities such as arterial hypertension, heart disease, diabetes, and chronic lung disease; young adults and children seem to have relatively mild disease and low mortality.^2,3^

To date, there are no proven, clinically effective pharmacological treatments against COVID-19, but multiple ongoing trials are evaluating novel and repurposed drugs.^4^ Among the repurposed drugs being rapidly investigated are the commonly used antimalarial and anti-inflammatory drugs chloroquine (CQ) and hydroxychloroquine (HCQ).^5^ These drugs have become the focus of global scientific, media, and political attention despite the lack of randomized controlled trials supporting their efficacy against COVID-19.^6^ Chloroquine has been used worldwide for about 75 years, and it is listed by the WHO as an essential medicine for malaria, whereas HCQ is widely used to treat autoimmune diseases such as systemic lupus erythematosus (SLE) and rheumatoid arthritis (RA).^7^ Both drugs have an established clinical safety profile,^8^ but their efficacy and safety for COVID-19 treatment or prevention remain to be defined.^9,10^

Chloroquine is a 4-aminoquinoline that was synthesized in Germany by Bayer in 1934 and emerged in the 1940s as an effective substitute for quinine, an antimalarial therapy used for centuries.^11^ Once a frontline drug for the treatment and prophylaxis of malaria, the efficacy of CQ was mostly lost because of the emergence of CQ-resistant *Plasmodium falciparum* strains in all endemic regions, including sub-Saharan Africa. Since about 2005, CQ has been replaced by artemisinin-based combination therapy to treat uncomplicated *P. falciparum* malaria across Africa, but it is still widely used to treat non-falciparum malaria, primarily outside of Africa.^12^ After CQ was found to have persistent immunomodulatory effects after cessation of short-term treatment, Winthrop developed and patented HCQ, which has an N-hydroxyl-ethyl side chain in place of the N-diethyl group, and therefore less tissue accumulation and a more favorable safety profile than CQ.^13,14^

There are rational arguments, preclinical evidence of activity, and long-term evidence of safety for other indications to justify CQ/HCQ trials for the treatment and prevention of COVID-19.^15^ Their mechanisms of action are incompletely understood but may include fusion and uncoating blockade,^17^ lysosomal alkalinization,^18^ interaction with the angiotensin-2 converting enzyme receptor,^19^ and immune modulation.^20^ However, in vitro antiviral activity of CQ/HCQ has not yet been translated into efficacy for any viral infection, and these drugs have been detrimental in some studies (e.g., for the treatment of chikungunya).^16^ Of note, for SARS-CoV-2, the in vitro activity of HCQ appears to be greater than that of CQ, which might allow for a lower dosage for HCQ.^21,22^ To date, the quality of available evidence for the clinical effectiveness of CQ/HCQ alone or in combination with other drugs (e.g., azithromycin) is low, because of small sample size, poorly defined clinical outcomes, and lack of randomization in published studies.^23–26^ Thus, the results of early clinical studies cannot yet be considered conclusive.

There remains an urgent need for high-quality evidence on the clinical value of CQ/HCQ alone or in combination with other drugs for the treatment of COVID-19. One global-scale effort is the ongoing WHO Solidarity trial, a large, adaptive, five-arm multinational (including South Africa) trial comparing four potential COVID-19 regimens: remdesivir, HCQ, lopinavir–ritonavir, and lopinavir–ritonavir plus interferon beta, all of which are compared with optimal supportive care, with in-hospital mortality as the primary end point. Secondary end points will be the duration of hospital stay and proportion of patients requiring intensive care unit admission or mechanical ventilation. The adaptive study design allows for dropping poorly performing arms and including additional promising therapeutics.^27^ Discovery is a component of the Solidarity trial, with identical arms and more complex end points, and is funded by the “Institut National de la Santé et de la Recherche Médicale,” France’s national health and medical research agency. More than 500 patients have already been enrolled in the Discovery trial, and preliminary analysis is ongoing. Also, the Recovery trial (for randomized evaluation) is a UK component of Solidarity, with more than 1,500 participants already enrolled.^27^ Also, CQ/HCQ Prevention of COVID-19 in the Healthcare Setting (COPCOV), a large (*n* = 40,000) multicentric trial in which participants will be randomized to receive either CQ or HCQ versus placebo, is being launched in Europe and Asia, and participation of African sites is being considered.^28^

Unfortunately, indiscriminate promotion of CQ/HCQ (with or without azithromycin) based on the aforementioned low-quality data for COVID-19 treatment has led to widespread shortages, self-use, and fatal overdoses.^29^ Chloroquine (and to a lesser extent HCQ) has been used for decades with few major safety issues at the usual antimalarial dosages in short-course regimens (2.5 g in 3 days for ≥ 60 kg adults).^30^ Although rare, cardiac toxicity (corrected QT interval [QTc] prolongation leading to torsades de pointes and ventricular fibrillation) is a serious, life-threatening complication, especially in patients with underlying cardiac disease, concurrent use of other drugs with QTc effect, or with supratherapeutic dosing.^26^ The therapeutic window is however larger with HCQ, which is mainly used in chronic administration for rheumatic disorders, usually at dosages of 200 to 400 mg/day in adults. The major toxicity of chronic CQ/HCQ use is retinopathy.^31^ Other important adverse effects associated with CQ/HCQ are listed in Table 1.^31–36^ Of great concern are frequent drug–drug interactions between CQ/HCQ and other medications used for prevalent chronic diseases in Africa, such as HIV infection and tuberculosis, and the concurrent use of antibiotics such as fluoroquinolones (Table 2).^37–44^ As an example, coadministration of azithromycin with CQ/HCQ should be cautiously approached and closely monitored because of additive risk for QTc prolongation and subsequent cardiac complications.^32^

**Table 1:** Main side effects of Chloroquine and Hydroxychloroquine^31–36^

System	Chloroquine	Hydroxychloroquine
Cardiovascular	QTc prolongation and cardiomyopathy	QTc prolongation and cardiomyopathy
Gastrointestinal	Nausea, vomiting, and abdominal pain	Nausea, vomiting, and abdominal pain
Dermatologic	Pruritis	Pruritis
Musculoskeletal	Myopathies and myasthenia-like syndromes	Sensorimotor disorders
Nervous	Seizures, tinnitus, and dystonia	Headache, dizziness, and tinnitus
Psychiatric	Depression and psychosis	Emotional lability
Ocular	Maculopathy and macular degeneration, and retinopathy	Blurred vision and retinopathy
Metabolic	Hypokalemia, hypercalcemia, and hypoglycemia	Hypoglycemia

**Table 2:** Drug interactions between CQ/HCQ and antituberculous or antiretroviral therapies

Medicine	Potential interaction with CQ/HCQ
Efavirenz	Limited clinical data. May increase (inhibition of CYP2C8) or decrease (induction of CYP3A4) exposure. Concurrent use may increase the risk of QT interval prolongation.
Lopinavir/ritonavir or atazanavir/ritonavir	Limited clinical data. May increase exposure by inhibition of CYPs 2C8, 3A4, and 2D6. Concurrent use may increase the risk of QTc interval prolongation.
Rifampicin	Limited clinical data. Induces phase-I and phase-II enzymes and transporters. Induction of CYP3A4 may decrease CQ/HCQ exposure.
Levofloxacin and moxifloxacin	Concurrent use may increase the risk of QTc interval prolongation.
Bedaquiline	Concurrent use may increase the risk of QTc interval prolongation.

CQ = chloroquine; HCQ = hydroxychloroquine. The metabolism of HCQ and CQ is predominantly mediated by the hepatic cytochrome P450 (CYP) enzymes 3A4 and 2D6, but 2C8 and 3A5 are also important. Any drug that induces or inhibits these CYP enzymes may potentially alter CQ/HCQ concentrations.^37–44^

Of note, *P. falciparum* resistance to CQ is widespread in sub-Saharan Africa, and artemisinin-based combination therapy has been the first-line treatment for uncomplicated malaria in all African countries for more than 10 years.^11^ Although currently inappropriate, widespread CQ/HCQ use for COVID-19 treatment or prevention should therefore have little impact on *P. falciparum* treatment outcomes. However, this may increase selection of resistance to CQ in *P. falciparum*, which has decreased in recent years, or in other *Plasmodium* species, for which CQ remains the treatment of choice.^45^ Furthermore, we call for caution regarding the widespread use of azithromycin (coadministered with CQ/HCQ) for treatment of COVID-19, as it may increase selection of bacterial resistance to this macrolide. In sub-Saharan Africa, azithromycin is an important treatment for bacterial infections including typhoid fever, especially where multidrug resistance (ampicillin, chloramphenicol, trimethoprim–sulfamethoxazole, and fluorquinolones) in *Salmonella typhi* is on the rise.^46,47^

Other concerns regarding the promotion of untested therapies for COVID-19 include fraud related to the growing market of substandard and falsified drugs^48^ and diversion of CQ/HCQ from other chronic conditions for which they are medically indicated, in particular SLE and RA.^49^ Safety issues are especially concerning for Africa because of relatively weak monitoring systems for off-label drug use and adverse events; these systems are robust in countries with strong national insurance schemes or with adequate private sector medical insurance. In addition, the promotion of CQ/HCQ for COVID-19 may lead to shortages and/or increased market prices of these medicines for malaria, SLE, and RA. One strategy to protect African countries from these threats is to leverage a collaborative network like the African Vaccine Regulatory Forum to coordinate cross-continental production, distribution chains, and post-marketing surveillance. Another model for quick, low-cost distribution of a COVID-19 drug or vaccine (once proven efficacious) would be to piggyback on platforms currently supported by the Global Fund, the U.S. President’s Emergency Plan for AIDS Relief, and other organizations. African countries should also establish and strengthen prescription-monitoring schemes to ensure that off-label use of any drug(s) is appropriate and beneficial in this pandemic. For example, in South Africa, prescribers are required to inform the regulatory agency about off-label use of existing drugs in COVID-19 treatment. This process will help gather information on treatment outcomes pending results from clinical trials.

Importantly, patients at risk of COVID-19 complications are also those most at risk of drug–drug interactions and drug-associated toxicity. These include the following patients: 1) older than 60 years (estimated at 10–20% of the African population)^50^; 2) with comorbidities, such as arterial hypertension (30% of African adults),^51^ diabetes (4% of African adults),^52^ chronic lung disease, malignancies, and immunosuppressive conditions; and 3) concurrently receiving medications with potential for drug interactions or additive toxicity. For these vulnerable populations, off-label CQ/HCQ use should be considered with the utmost care, ideally following monitored research protocols in hospital and outpatient settings.

In conclusion, there is currently no evidence that CQ or HCQ, two low-cost drugs for which we have extensive experience for treatment of malaria and rheumatic disorders, has beneficial effects on the clinical course of COVID-19 patients. There are more than 80 ongoing trials of CQ or HCQ, used alone or in combination with a variety of other drugs registered on ClinicalTrials.gov. The results of these studies, including Solidarity and its companion trials (Discovery and Recovery) as well as COPCOV are eagerly awaited. Meanwhile, the off-label use of CQ and HCQ to prevent or treat COVID-19 in Africa and elsewhere must be viewed with greatest caution, considering potential serious toxicities and benefit versus risk. If the effectiveness of these and other drugs is established in global trials, therapeutics for COVID-19 will require further operational evaluation in Africa.
